# Prognostic Impact of Serum Albumin Levels at Diagnosis in Patients with Chronic Lymphocytic Leukemia

**DOI:** 10.3390/jcm15031315

**Published:** 2026-02-06

**Authors:** Selin Küçükyurt Kaya, Hacer Berna Afacan Öztürk, Oguzhan Koca, Lale Aydın Kaynar, Ufuk Gördük, Asena Dikyar, Haktan Bağış Erdem, Kadir Acar, Murat Albayrak, Ahmet Kürşad Güneş

**Affiliations:** 1 Department of Hematology, Ankara Etlik City Hospital, 06170 Ankara, Türkiye; bernaafacan@yahoo.com (H.B.A.Ö.); drlaleaydin@hotmail.com (L.A.K.); m.ufukgorduk@hotmail.com (U.G.); asenadikyar@gmail.com (A.D.); acarkadir@yahoo.com (K.A.); muratalbayrak71@yahoo.com (M.A.); ahmetkgunes@gmail.com (A.K.G.); 2 Department of Internal Medicine, Cerrahpaşa Faculty of Medicine, İstanbul University-Cerrahpaşa, 34098 İstanbul, Türkiye; ogkoca@gmail.com; 3 Department of Medical Genetics, Ankara Etlik City Hospital, 06170 Ankara, Türkiye; haktanbagis.erdem@sbu.edu.tr

**Keywords:** chronic lymphocytic leukemia, prognosis, albumin, survival

## Abstract

**Background/Objectives**: Chronic lymphocytic leukemia (CLL) displays substantial clinical heterogeneity, yet access to genomic prognostic testing remains limited in many real-world and resource-constrained settings. Readily available biomarkers that reflect disease biology are therefore clinically valuable. Serum albumin, an inexpensive marker associated with systemic inflammation and tumor burden, has shown emerging prognostic potential. This study evaluated the impact of baseline albumin on time to first treatment (TTFT) and overall survival (OS) in CLL. **Methods**: We retrospectively analyzed adult patients with confirmed CLL treated at a single tertiary center. Baseline demographic, clinical, and laboratory features were recorded, and serum albumin was dichotomized at 4 g/dL. TTFT and OS were estimated using the Kaplan–Meier methodology. Variables with *p* < 0.1 in univariate analyses were included in multivariate Cox regression models. **Results**: A total of 230 patients were included. The median age at diagnosis was 62.5 years; 52.2% were male, and 14.8% had serum albumin <4 g/dL. Low albumin was associated with older age, advanced Rai/Binet stage, anemia, higher lymphocyte counts, and greater treatment requirement (all *p* < 0.05). Median follow-up was 20 months (range, 1–288). Patients with albumin <4 g/dL had inferior 5-year OS (78.4% vs. 98.7%). Although serum albumin correlated with both TTFT and OS in univariate analyses, it did not remain independently significant in multivariate models. **Conclusions**: While not independently prognostic, baseline serum albumin is strongly linked to adverse clinical features and poorer unadjusted survival. As a readily available, low-cost parameter, albumin may offer practical value for early risk stratification—particularly in regions where routine molecular testing is constrained.

## 1. Introduction

Chronic lymphocytic leukemia (CLL) is the most common leukemia in adults and is characterized by a highly heterogeneous clinical course [1]. While many patients experience an indolent disease requiring prolonged observation without therapy, others develop a more aggressive clinical course marked by rapid disease progression and early need for treatment initiation. Consequently, accurate prediction of the disease’s biological behavior at diagnosis is essential for optimizing treatment timing, tailoring follow-up strategies, and improving patient outcomes [1].

Traditionally, prognostic assessment in CLL has relied on the Rai and Binet staging systems, which are simple, inexpensive, and widely applicable tools based on physical examination findings and routinely available hematologic parameters [2,3,4]. These classifications stratify patients into low-, intermediate-, and high-risk categories and have long guided clinical decision making, particularly with regard to treatment initiation. While symptomatic or high-risk patients generally require therapy, treatment selection is further influenced by patient-related factors such as comorbidities and functional status, as well as established genomic risk features [1]. Nevertheless, despite their continued clinical utility, the Rai and Binet systems provide limited insight into disease behavior and may fail to adequately distinguish patients with early-stage disease at risk of more rapid progression.

Over the past two decades, advances in molecular and cytogenetic profiling have markedly improved prognostic stratification in CLL. Established risk factors include TP53 disruption (TP53 mutations and/or deletion 17p), immunoglobulin heavy-chain variable region (IGHV) mutation status, complex karyotype, and depth of response assessed by measurable residual disease (MRD) [1,5]. These parameters are incorporated into validated prognostic models, most notably the CLL-International Prognostic Index (CLL-IPI) and increasingly inform frontline treatment selection in the era of targeted therapies [1,6,7]. Nevertheless, access to comprehensive molecular testing remains variable across institutions, and limitations related to cost, technical infrastructure, and turnaround time restrict its routine implementation in real-world clinical practice, particularly in resource-limited settings.

In this context, there is ongoing interest in identifying simple, inexpensive, and universally available laboratory parameters that may complement established prognostic tools. Serum albumin, a routinely assessed biochemical marker, reflects several clinically relevant processes, including systemic inflammation, nutritional status, hepatic function, and overall disease burden. Hypoalbuminemia has been consistently associated with inferior survival outcomes across a wide range of solid tumors and hematologic malignancies, including aggressive lymphomas, multiple myeloma, and acute leukemias. In CLL, low serum albumin may reflect increased tumor burden, inflammatory cytokine activation, and immune dysregulation [8,9,10,11]. Nevertheless, evidence regarding its prognostic significance in CLL remains limited.

This study aimed to assess the impact of baseline serum albumin levels on time to first treatment (TTFT) and overall survival (OS) in patients with CLL, highlighting its role as a simple and widely accessible prognostic biomarker.

## 2. Materials and Methods

### 2.1. Study Design and Patient Population

This retrospective, single-center, observational study included patients aged ≥ 18 years who were diagnosed with CLL according to the International Workshop on CLL–National Cancer Institute (iwCLL-NCI) criteria [12], between January 2010 and November 2024 and who continued follow-up at our center. The study protocol was approved by the local ethics committee.

Patients with conditions known to cause hypoalbuminemia—including active infection, chronic inflammatory disease, chronic liver disease, or nephrotic syndrome—were excluded.

### 2.2. Data Collection

Data were retrospectively extracted from the electronic medical record system and archived paper charts of patients routinely followed at our center. Demographic and clinical characteristics; date of diagnosis; Rai and Binet stages [2,3]; laboratory parameters including serum albumin, leukocyte count, absolute lymphocyte count, platelet count, hemoglobin and β2-microglobulin levels; cytogenetic and molecular findings (when available); most recent follow-up date; and survival status were collected.

Baseline serum albumin was defined as the albumin level measured at the time of diagnosis or within ±30 days of the diagnosis date, before the initiation of any CLL-directed therapy. When multiple measurements were available within this time window, the value closest to the diagnosis date was used for analysis.

Serum albumin was measured using an automated analyzer in the institutional central laboratory as part of routine clinical chemistry testing. Albumin levels were reported in g/dL, in accordance with standard laboratory practice. Because serum albumin is a widely used and standardized biochemical parameter, assay-specific variability is not expected to materially affect the results.

### 2.3. Study Objectives

The primary objective of this study was to evaluate the prognostic impact of baseline serum albumin levels at diagnosis on TTFT and OS in patients with CLL. A secondary objective was to explore the association between baseline serum albumin levels and established adverse clinical and laboratory features.

### 2.4. Statistical Analysis

All statistical analyses were performed using IBM SPSS Statistics for Windows, version 25.0 (IBM Corp., Armonk, NY, USA). Nominal variables were summarized as frequencies and percentages. Categorical variables were compared using Pearson’s chi-square test or Fisher’s exact test, as appropriate. Continuous variables were non-normally distributed and were therefore presented as median values with ranges (minimum–maximum). The Mann–Whitney U test was used to compare continuous variables between groups.

Survival distributions were estimated using the Kaplan–Meier method. TTFT was defined as the time from diagnosis to the initiation of first-line therapy. OS was defined as the time from diagnosis to death from any cause or last follow-up. Cutoff values for key variables were determined as follows:

Age was dichotomized at 65 years, which corresponded to the optimal threshold identified by receiver operating characteristic (ROC) analysis and is commonly used for risk stratification in CLL [13]. Eastern Cooperative Oncology Group (ECOG) performance status was categorized as 0–2 versus 3–4, based on ROC analysis and the clearer separation of survival curves observed on Kaplan–Meier analysis [14]. Hemoglobin levels were dichotomized at 11 g/dL, consistent with the Rai staging definition of anemia and supported by ROC-derived optimal cutoff values. Serum albumin was dichotomized at 4 g/dL, a clinically meaningful threshold widely used in hematologic malignancies, and further supported by ROC analysis in this cohort.

Variables associated with OS at a significance level of *p* < 0.1 in univariate analyses were included in a multivariate Cox proportional hazards model. A stepwise selection procedure was applied to identify independent prognostic factors, and results were expressed as hazard ratios (HRs) with corresponding 95% confidence intervals (CIs). All statistical tests were two-sided, and *p* < 0.05 was considered statistically significant. Cytogenetic and molecular data were available for a limited proportion of patients and were therefore not included in the univariate or multivariate analyses to avoid potential bias and misleading results.

## 3. Results

A total of 230 patients with CLL were included in this study. The median age at diagnosis was 62.5 years (range, 29–93), and 52.2% of the cohort were male (Table 1). Overall, 42.2% of patients were aged ≥ 65 years, and most patients had an ECOG performance status of ≤2 (81.7%). Demographic, clinical, and laboratory characteristics of the cohort are summarized in Table 1.

At diagnosis, 83.9% of patients were classified as early-stage according to the Rai system, and 90.4% were early-stage based on the Binet staging system. The ratio of patients with del (17p)/TP53 mutation and unmutated IGHV was 2.6% and 3.9%, respectively.

**Figure 1:**
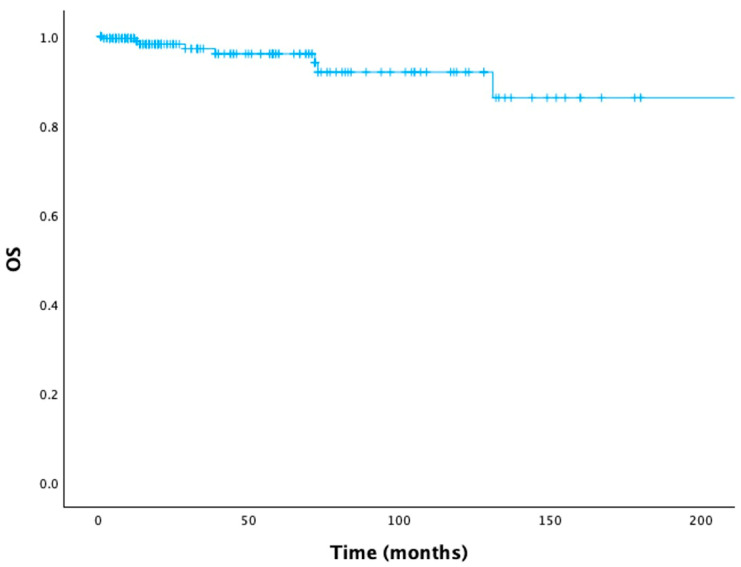
Overall survival curve for the entire cohort (OS, overall survival).

The median follow-up duration was 20 months (range, 1–288 months), with eight OS events (deaths) recorded among the 230 patients. The OS curve for the entire cohort is shown in Figure 1. The 5-year OS rate for all patients was 96.1%. Among patients who required therapy (17.8%), first-line treatment consisted of chemoimmunotherapy in 78.0%, BTK inhibitors in 14.6%, and a BCL-2 inhibitor plus anti-CD20 monoclonal antibody in 7.3%.

The median serum albumin level for the entire cohort was 4.4 g/dL (range, 2.3–5.3). Serum albumin was ≥4 g/dL in 85.2% of patients (N = 196). Baseline demographic and clinical characteristics stratified by albumin level (<4 vs. ≥4g/dL) are presented in Table 1. Patients with serum albumin <4 g/dL were significantly older and had lower median hemoglobin and higher median lymphocyte counts (Table 1). Higher Rai and Binet stages were also more frequent among patients with low serum albumin, and treatment requirement was significantly more common in this subgroup (all *p* < 0.05).

**Figure 2:**
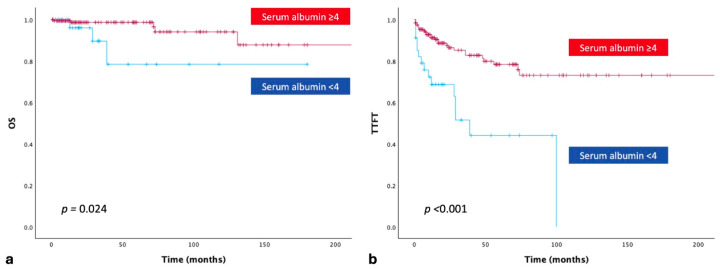
Comparison of CLL patients with baseline serum albumin levels < 4 g/dL and ≥ 4g/dL regarding overall survival (**a**) and time to first treatment (**b**) (OS, overall survival; TTFT, time to first treatment).

The 5-year OS rates for patients with serum albumin ≥ 4 g/dL and <4 g/dL were 98.7% and 78.4%, respectively. Patients with serum albumin ≥ 4 g/dL had significantly longer OS than those with serum albumin < 4 g/dL (*p* = 0.024; Figure 2a). When baseline serum albumin levels were compared between treatment-naïve and treated patients, serum albumin < 4 g/dL was significantly associated with an increased need for therapy (*p* = 0.002; Table 1). As expected, TTFT was significantly inferior among patients who required treatment (*p* < 0.001; Figure 2b).

**Figure 3:**
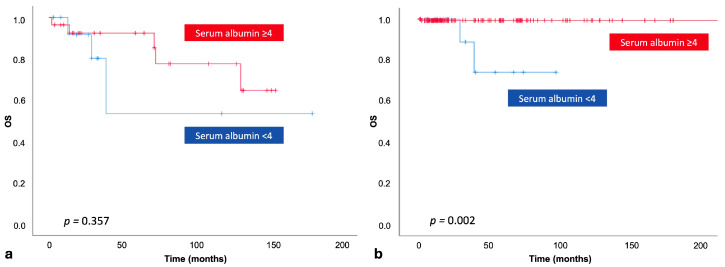
Comparison of overall survival between patients with baseline serum albumin levels < 4 g/dL and ≥4 g/dL in the treated subgroup (**a**) and the treatment-naïve subgroup (**b**).

Among patients who required treatment (N = 41), 31.7% had serum albumin levels < 4 g/dL (N = 13). Within this subgroup, OS did not differ significantly between patients with serum albumin ≥ 4 g/dL and those with <4 g/dL (*p* = 0.357; Figure 3a). In contrast, among treatment-naïve patients (N = 189), OS was significantly worse in those with serum albumin < 4 g/dL (*p* = 0.002; Figure 3b).

In univariate analyses, several baseline factors demonstrated a significant impact on TTFT, including age at diagnosis, ECOG performance status, hemoglobin and platelet counts, and Rai/Binet stages. For OS, age, ECOG performance status, hemoglobin level, Rai stage, and the need for treatment showed significant prognostic influence (all *p* < 0.05). Baseline serum albumin also showed a clear effect on both TTFT and OS in univariate analysis; however, this effect did not persist after adjustment for other variables in multivariate modelling (all *p* < 0.05; Table 2). In the multivariate model, ECOG performance status, hemoglobin level, and platelet count remained the only independent determinants of TTFT (Table 2).

## 4. Discussion

In our retrospective cohort, we demonstrated that baseline serum albumin—an inexpensive, routinely available biochemical parameter—has meaningful prognostic relevance in patients with CLL. Although albumin did not remain an independent marker in multivariate analysis, its strong associations with advanced clinical stage, higher disease burden, treatment requirement, and inferior unadjusted survival metrics highlight its value as a readily measurable surrogate of host–tumor interactions.

Hypoalbuminemia has been associated with adverse outcomes across solid tumors and hematologic malignancies, reflecting systemic inflammation, impaired nutritional status, and cytokine-driven catabolism [6,7,8]. Similar observations have been reported in CLL through inflammation-based indices integrating albumin, including the C-reactive protein-to-albumin ratio (CAR) and albumin-to-fibrinogen ratio (AFR) [11,15]. Tang et al. demonstrated that CAR independently predicted both TTFT and OS in newly diagnosed CLL and improved the discriminatory capacity of the CLL-IPI [11]. Likewise, Zou et al. showed that AFR was an independent adverse prognostic factor regardless of IGHV status, β2-microglobulin level, or TP53 alterations [15]. These studies collectively indicate that albumin is not merely a routine biochemical parameter but a sensitive marker reflecting systemic inflammation and metabolic alterations that are closely associated with CLL biology.

Our findings extend this body of evidence by demonstrating that serum albumin alone—without combination into composite inflammatory indices—has significant univariate associations with both TTFT and OS, particularly among treatment-naïve patients. The marked survival gap between albumin ≥ 4 g/dL and <4 g/dL in this subgroup suggests that albumin-based risk stratification may be particularly informative in early-disease settings, where traditional high-risk genetic lesions may be absent or untested. Notably, in our cohort, the prognostic effect of serum albumin diminished once patients required therapy, likely because subsequent survival becomes dominated by disease refractoriness, treatment selection, and genetic complexities such as TP53 disruption, as illustrated in contemporary prognostic meta-analyses [7,14].

The lack of independent significance of serum albumin in multivariate modelling warrants careful interpretation and is most plausibly explained by confounding and collinearity with established adverse disease features. In our cohort, baseline serum albumin showed strong correlations with advanced Rai and Binet stage, anemia, and thrombocytopenia—parameters that reflect disease burden and remain independent predictors in our final model. These findings indicate that baseline serum albumin consistently identifies patients with higher disease burden and adverse clinical profiles at diagnosis, thereby providing clinically meaningful prognostic information even when not retaining statistical independence in multivariate modelling. Accordingly, the prognostic signal observed in univariate analyses should be interpreted as context-dependent, supporting the role of serum albumin as a clinically meaningful complementary prognostic marker, particularly in early-stage and treatment-naïve patients.

In addition, the limited availability of TP53, IGHV, and cytogenetic testing under real-world conditions in Türkiye may have constrained comprehensive risk characterization within our cohort; consequently, more extensive molecular profiling could potentially alter the strength or independence of the observed association between serum albumin and clinical outcomes.

Although numerous prognostic models have been developed to refine risk stratification in CLL, their applicability in the modern therapeutic landscape has become increasingly complex. Several widely used scores—such as the CLL1 prognostic model (CLL1-PM) [16], which incorporates del(17p), del(11q), unmutated IGHV, serum β2-microglobulin, lymphocyte doubling time, and age; the International Prognostic Score for Early-stage CLL (IPS-E) [17], which includes unmutated IGHV, lymphocyte count, and palpable lymphadenopathy; and the CLL-IPI [6], which integrates age, clinical stage (Rai/Binet), β2-microglobulin, IGHV mutation status, and TP53 disruption—were all designed to predict TTFT and long-term outcomes by combining multiple independent prognostic variables. However, a major limitation of these models is their heavy reliance on genetic and molecular parameters, which may not be routinely available in many real-world settings, including ours. Moreover, with the advent of targeted therapies, the prognostic impact of individual variables within these models has become less straightforward. For instance, although unmutated IGHV and detectable MRD predict inferior outcomes following ibrutinib–venetoclax combinations, patients with mutated IGHV—despite less favorable MRD responses—continue to demonstrate superior progression-free survival. This paradox illustrates how established prognostic factors may lose discriminatory capacity or exert opposing effects in the context of novel therapeutic agents [18]. Collectively, these challenges underscore the need for simple, accessible, and treatment-agnostic prognostic markers. In this regard, our findings suggest that serum albumin remains a clinically meaningful indicator of disease burden and treatment requirement, particularly valuable in settings where comprehensive molecular profiling cannot be universally implemented.

### Study Limitations

Our study has several limitations. The retrospective design, relatively small sample size, and limited follow-up duration may restrict the generalizability of the findings. In addition, due to current regulatory constraints in Türkiye, key molecular tests such as IGHV mutation analysis, del(17p), and TP53 mutation status are not routinely performed in treatment-naïve patients without an indication for therapy. This limitation constrained our ability to incorporate comprehensive genomic risk factors into multivariate models and may have influenced the interpretation of serum albumin within a fully characterized risk framework.

## 5. Conclusions

In conclusion, our findings demonstrate that baseline serum albumin is a clinically meaningful and readily available prognostic marker in patients with CLL. Although serum albumin did not retain independent prognostic significance in multivariate analyses, low albumin levels at diagnosis were consistently associated with advanced clinical stage, higher disease burden, increased treatment requirement, and inferior unadjusted OS. Notably, the prognostic impact of serum albumin was most pronounced in treatment-naïve patients, suggesting particular utility in early-stage disease where conventional high-risk genetic markers may be absent or unavailable.

Given its low cost, universal availability, and ease of measurement, serum albumin represents a practical adjunct to existing prognostic tools, particularly in real-world settings with limited access to comprehensive molecular profiling. While it should not be considered a substitute for established genomic risk factors, baseline serum albumin may help refine initial risk assessment, identify patients who warrant closer surveillance, and support clinical decision making at diagnosis.

Prospective studies with longer follow-up and integrated molecular characterization are warranted to further clarify the role of serum albumin within contemporary prognostic frameworks and to determine whether its incorporation into existing or novel risk models may enhance risk stratification in the era of targeted therapies.

## Figures and Tables

**Table 1:** Demographic features and characteristics of patients (ECOG, Eastern Cooperative Oncology Group Performance Status; IGHV, immunoglobulin heavy chain variable region). A *p*-value < 0.05 was considered statistically significant.

Characteristics	Entire Cohort (N = 230)	Serum Albumin ≥4 g/dL (N = 196)	Serum Albumin <4 g/dL (N = 34)	*p* Value
**Sex, n (%)**				
Female Male	110 (47.8) 120 (52.2)	95 (48.5) 101 (51.5)	15 (44.1) 19 (55.9)	0.389
**Age at diagnosis, years**				
Median (Range)	62.5 (29–93)	61 (29–86)	69 (42–93)	**0.001**
**Age categories, n (%)**				
<65 years ≥65 years	133 (57.8) 97 (42.2)	123 (62.8) 73 (37.2)	10 (29.4) 24 (70.6)	**<0.001**
**ECOG, n (%)**				
0-1-2 3–4	188 (81.7) 42 (18.3)	169 (86.2) 27 (13.8)	19 (55.9) 15 (44.1)	**<0.001**
**Hemoglobin (g/dL)**				
Median (Range)	13.4 (5.7–18.3)	13.7 (7.6–18.3)	10.6 (5.7–15)	**<0.001**
**White blood count, ×10^9^/L**				
Median (Range)	22.1 (7.55–536.3)	21.14 (7.55–274)	35 (8.9–536.3)	**0.005**
**Absolute lymphocyte count, ×10^9^/L**				
Median (Range)	15.95 (5–522.97)	15.45 (5–252.22)	31.06 (5.23–522.97)	**0.003**
**Platelet count (range), ×10^9^/L**				
Median (Range)	222 (11–489)	224 (12–489)	184.5 (11–345)	**0.023**
**Serum ß2 microglobulin, n (%)**				
≤3.5 mg/L >3.5 mg/L N/A	72 (31.3) 39 (17) 119 (51.7)	65 (33.2) 30 (15.3) 101 (51.5)	7 (20.6) 9 (26.5) 18 (52.9)	0.054
**Rai, n (%)**				
0-I-II III–IV	193 (83.9) 37 (16.1)	179 (91.3) 17 (8.7)	14 (41.2) 20 (58.8)	**<0.001**
**Binet, n (%)**				
A-B C	208 (90.4) 22 (9.6)	189 (96.4) 7 (3.6)	19 (55.9) 15 (44.1)	**<0.001**
**Del(17p)/TP53 mutation**				
Positive Negative N/A	6 (2.6) 36 (15.7) 188 (81.7)	6 (3.1) 23 (11.7) 167 (85.2)	0 (0) 13 (38.2) 21 (61.8)	0.091
**IGHV mutation status**				
Mutated Unmutated N/A	27 (11.7) 9 (3.9) 194 (84.3)	18 (9.2) 6 (3.1) 172 (87.7)	9 (26.5) 3 (8.8) 22 (64.7)	0.65
**Patients who required treatment**	41 (17.8)	28 (14.3)	13 (38.2)	**0.002**

**Table 2:** Univariate and multivariate analyses of treatment-free and overall survival of the entire cohort. * ß2 microglobin level available in 111 patients in the cohort (ECOG, Eastern Cooperative Oncology Group Performance Status; HR, hazard ratio; OS, overall survival; TTFT, time to first treatment). A *p*-value <0.05 was considered statistically significant.

		Univariate		Multivariate		Univariate		Multivariate	
Characteristics	Variables	TTFT-HR	*p* Value	TTFT-HR	*p* Value	OS-HR	*p* Value	OS-HR	*p* Value
Age at diagnosis,	<65								
years	≥65	1.97 (1.08–3.61)	**0.028**			16.01 (1.94–132.33)	**0.01**		
Sex	Female								
	Male	1.47 (0.80–2.71)	0.214			2.27 (0.52–9.91)	0.278		
ECOG	0–2								
	≥3	4.48 (2.45–8.19)	**<0.001**	2.93 (1.51–5.69)	**0.001**	14.53 (2.92–72.33)	**0.001**		
Hemoglobin	≥11								
(g/dL)	<11	5.74 (3.11–10.58)	**<0.001**	3.37 (1.62–6.99)	**0.01**	8.83 (2.10–37.03)	**0.003**		
Platelet count	≥100								
×10^9^/L	<100	11.75 (5.23–26.40)	**<0.001**	3.45 (1.35–8.82)	**0.001**	3.74 (0.45–31.36)	0.225		
ß2 microglobulin	≤3.5								
(mg/L), N = 111 *	>3.5	1.93 (0.88–4.23)	0.102			2.93 (0.49–17.54)	0.240		
Rai	0-I-II								
	III-IV	6.59 (3.59–12.10)	**<0.001**			8.32 (1.98–34.92)	**0.004**		
Binet	A-B								
	C	7.36 (3.79–14.29)	**<0.001**			4.74 (0.90–24.83)	0.066		
Treatment	Naive								
	Received					4.92 (1.15–21.03)	**0.032**		
Serum albumin	≥4								
(g/dL)	<4	3.75 (1.99–7.05)	**<0.001**			4.60 (1.07–19.71)	**0.040**		

## Data Availability

The data supporting the findings of this study are available from the corresponding author upon reasonable request.
